# Dual paths of information behavioral choice influenced by information and emotion: The moderating effect of user credibility

**DOI:** 10.1371/journal.pone.0353608

**Published:** 2026-07-10

**Authors:** Na Wang, Yaming Zhang, Yaya Hamadou Koura

**Affiliations:** 1 School of Economics and Management, Yanshan University, Qinhuangdao, China; 2 Development of Center for Internet Plus and Industry, Yanshan University, Qinhuangdao, China; 3 School of Foreign Languages, Yanshan University, Qinhuangdao, China; Dong-A University College of Business Administration, KOREA, REPUBLIC OF

## Abstract

In public opinion events, social media is often used as an important channel for the public to collect, process, and share information, and the topics and information on social media stimulates people to behave in creating related contents. This study integrates the elaboration likelihood model (ELM) to examine how information topics (central cue) and emotional responses (peripheral cue) shape user information behavior (likes, comments, forwards), and how user credibility moderates these effects. 56,663 Weibo posts (August 22 to September 5, 2023) about “Japan’s announcement of nuclear contaminated water discharge” were used for empirical analysis. Topics were identified via Latent Dirichlet Allocation (LDA), yielding four categories: Japanese aquatic products trade (Topic 1, 1.850%), seawater blackening (Topic 2, 8.822%), radioactivity exceedance (Topic 3, 13.286%), and global impact (Topic 4, 76.043%). Seven emotions were scored using the Dalian University of Technology affective lexicon. User credibility was coded from verification type. Baseline regression controlling for richness, mention, hashtags, URL, and text length showed that information topics significantly increased likes (β range: 0.164 to 0.333, p < 0.001), comments (β range: 0.063 to 0.138, p < 0.001), and forwards (β range: 0.083 to 0.156, p < 0.001), with the largest effects on likes. For emotions, anger, disgust, good and surprise positively influenced information behaviors (β range: 0.004 to 0.060, p < 0.01 to p < 0.001), while sadness, happy and fear showed negative or non‑significant effects. User credibility positively moderated all these relationships, with interaction β values ranging from 0.010 to 0.066 for topics and from 0.006 to 0.040 for emotions. Likes were the most influenced behavior, followed by forwards, then comments. The study demonstrates that information topics and emotions, moderated by user credibility, drive distinct information behaviors. Authorities should leverage high‑credibility users, highlight impactful topics, and monitor emotional tones, especially negative ones, to guide public opinion effectively.

## 1. Introduction

Across various cultural contexts, social media has replaced the original means of communication such as newspapers, television, and radio and become an important medium for information dissemination and emotional expression [1]. People have become accustomed to collecting and sharing information on social media platforms, and adept at using the social media functions to express their personal views and attitudes about the existing information. For example, Facebook, Twitter, and Weibo have penetrated into people’s daily life and become the largest carrier of public opinion today [2]. Users’ information behavior in social media reflects their motivations, preferences and decision-making logic. Conducting an in-depth study of users’ information behavior can provide precise insights into users’ information needs, providing crucial evidence for relevant institutions to formulate public opinion response strategies.

Existing studies has extensively explored the factors influencing users’ information behavior from multiple perspectives, including information characteristics, linguistic features and user characteristics. Studies have found that numerous factors, such as information topic, information quality, emotional expression, rhetorical strategies and user gender, influence can users’ engagement with information dissemination [3–7]. Among these, two factors have been consistently identified as key drivers: information topic and emotional response. Information topics can reflect the degree to which the content matches users’ cognitive needs and situational contexts, prompting systematic processing of the message. Emotional response, on the other hand, captures the affective intensity triggered by the content, which often leads to heuristic and impulsive responses [8,9]. Empirical studies have shown that both factors independently and interactively shape users’ engagement behaviors [6]. Consequently, the ELM theoretical framework, which integrates cognitive and affective pathways, is widely used to explain how users process information and make behavioral decisions on social media.

The elaboration likelihood mode (ELM) categorizes the information processing process into two possible paths based on differences in information content and individual information processing ability [10,11]. Specifically, when people demonstrate both high motivation to process information and sufficient cognitive capacity, they are more likely to engage in central route processing by systematically analyzing information content [12]. In contrast, when either motivation or cognitive capacity is constrained, peripheral factors such as intuition or emotion become the basis for people’s judgments [13–15]. This dual-pathway framework provides a refined model for analyzing user interactions on social media platforms, offering deeper insights into users’ attitudes and behavioral patterns.

Social media platforms provide people a variety of interactive features, including but not limited to liking, commenting, and forwarding [16]. These distinct engagement patterns reflect different processes of information processing. According to ELM, central route processing involves effortful cognitive elaboration, which is more likely to generate commenting behavior that requires articulating one’s own opinions. Peripheral route processing relies on heuristic cues such as emotional impulses, which is more likely to generate liking behavior as a quick, low-effort response. Forwarding lies between the two, as it can be driven by either emotional arousal or cognitive evaluation of information value [17,18]. Crucially, the combinatorial configurations of information attributes differentially trigger specific information behaviors, and each possessing unique potential to shape the trajectory of public opinion dissemination.

While ELM provides a useful framework for understanding information processing, how its central and peripheral routes specifically map onto different information behaviors has not been rigorously tested. Moreover, empirical evidence on how specific emotional dimensions differentially influence these behaviors is still limited, with many studies relying on coarse positive-negative sentiment classifications. To address these gaps, this study focuses on the Weibo, an important platform for generating and disseminating public opinion in China, and selects the major event of Japan’s announcement of nuclear contaminated water discharge for empirical analysis. On August 22, 2023, the Japanese government officially announced its decision to discharge nuclear contaminated water from the Fukushima Daiichi Nuclear Power Plant into the Pacific Ocean on August 24. This announcement sparked widespread public attention and discussion around the world, the Chinese public was no exception. Based on the Weibo data collected related to this event, the study extracted information topics and emotional responses as independent variables and empirically tested their impact on likes, comments, and forwards. In addition, considering the heterogeneity of users in social media platforms, this study discussed the moderating effect of user credibility.

## 2. Literature review

### 2.1. Information and information behavior

According to ELM, information content is the main cue for people to process information. If people think that information can meet their needs, they will take appropriate information behavior [19]. However, not all information content exerts the same influence. Different information topics vary in their ability to capture attention and drive behavioral responses [20]. Information adoption model posits that information usefulness, which is partly determined by topic relevance, serves as a key antecedent of information adoption and subsequent sharing behaviors [14]. When a topic is perceived as highly relevant to one’s concerns or values, it increases the perceived utility of engagement, thereby motivating behavioral responses such as liking, commenting, or forwarding [14,21]. Specifically, attractive topics can bring users a sense of freshness, and increase their participation [22]. For example, during the 2020 floods in China’s Yangtze River basin, topics in the disaster response and recovery phases have a strong public appeal [23]. In the case of the 7.20 Henan rainstorm and Typhoon Mangkhut in China, information on disaster impacts and relief operations is the main focus of communication [24,25]. Such topic salience fundamentally regulates information diffusion scope and societal impact through cognitive prioritization mechanisms [26].

In this study, information topic refers to the core issue discussed in a social media post, identified through text mining. Within this ELM-based framework, an information topic as a key factor shaping users’ cognitive evaluation. Topics that are closely related to the core event or carry significant personal or social implications are more likely to attract users’ attention, enhance their motivation to process information, and ultimately trigger behavioral responses [4]. Considering that there is more than one information behavior that people can adopt during the process of public opinion dissemination, the following hypotheses are proposed.

H1a -H1c Information topics demonstrating higher relevance strength to public opinion events exhibit a greater propensity to motivate user liking, commenting and forwarding behaviors.

### 2.2. Emotion and information behavior

Emotion is regarded as a crucial incentive factor during the emergence of information behavior [27,28]. Emotion can affect users’ search intention, search strategy, search performance, and search frequency and also drive other information behaviors, such as information expression, information sharing, and possibly derived information exchange behaviors and decision-making [29–32]. According to ELM, emotion provides a peripheral route for information processing: when users lack the motivation or cognitive capacity for systematic elaboration, they rely on emotional cues to make rapid judgments and take actions [3,15]. Within the social media environment, this peripheral mechanism translates into distinct patterns of engagement.

Specifically, liking is the most direct emotional response. When a post triggers strong emotions, users are inclined to click “like” as an immediate expression of their affective state [33]. When posts convey positive emotions, they tend to exhibit high similarity and concentrated dissemination, making them more likely to be noticed and forwarded by official media [29,34]. When posts carry negative emotions, they are often perceived as exaggerated and provocative, which tends to attract more public commenting [20,35]. Furthermore, after further refining the types of emotions, scholars found that disgust and anger can promote users to retweet, while fear and sadness may prevent information dissemination [36,37].

However, controversy still exists about the role of different emotions on information dissemination, and the specific effects of different emotions on liking, commenting, and forwarding remain to be clarified. In this study, emotional response is defined as the user’s affective reaction to a post, operationalized by continuous scores of seven emotion types (anger, disgust, fear, good, sadness, surprise, and happy). Considering the complex emotional responses of people during the public opinion dissemination, the following hypotheses are proposed.

H2a-H2g Information with anger/disgust/fear/good/sadness/surprise/happy emotion is more likely to be liked

H3a-H3g Information with anger/disgust/fear/good/sadness/surprise/happy emotion is more likely to be commented.

H4a-H4g Information with anger/disgust/fear/good/sadness/surprise/happy emotion is more likely to be forwarded.

### 2.3. The moderating role of user credibility

Social media platforms are flooded with extreme, distorted, and deliberately misleading or inflammatory content, making it difficult for users to make information behavioral decisions based solely on information content or emotional responses. At this time, user credibility, defined as the degree to which a social media user is perceived by others as trustworthy, authoritative, and reliable become a critical moderating factor that affects people ‘s attitudes and behavioral tendencies [38]. According to the source credibility theory, information from high-credibility sources is more likely to be accepted and acted upon, especially when individuals lack the motivation or ability to process information systematically [39,40]. Similarly, the heuristic-systematic model posits that people often rely on credibility as a peripheral cue to form judgments quickly, particularly when navigating information overload [41].

In social media, people often judge the credibility of information by user credibility. Due to the difference of credibility and influence, the influence scope and dissemination effect of information published by different users are very different [42,43]. In general, the higher the user credibility, the more the authenticity of the information can be guaranteed [44]. When receiving information released by highly credibility users, people are more likely to believe and generate willingness to act [5,45,46]. To be sure, analyzing user credibility helps to predict and explain people’s behavioral patterns in the process of public opinion dissemination. However, the specific role of user credibility on different topics and emotions still needs to be further explored. Therefore, the following hypotheses are proposed.

H5a -H5c User credibility moderates the effect of information topic on liking, commenting and forwarding behaviors.

H5d -H5f User credibility moderates the effect of emotion on liking, commenting and forwarding behaviors.

**Fig 1:**
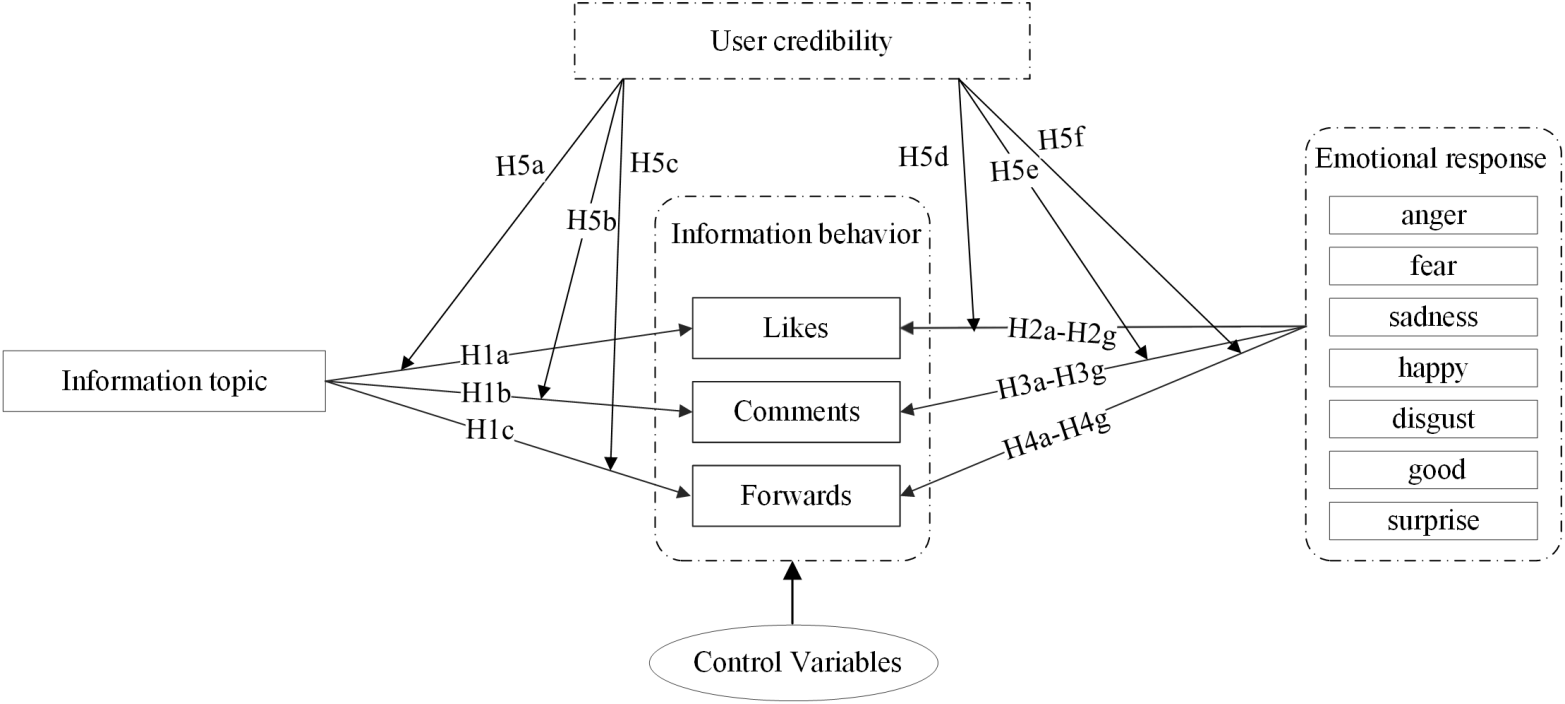
The research model.

In the approach, the information topic and emotional response were taken as independent variables, likes, comments, and forwards as dependent variables, and user credibility was selected as a moderating variable to explore public opinion participation decision-making. Combined with the above analysis, the overall analytical framework was drawn as shown in Fig 1.

## 3. Methods

### 3.1. Data collection

On Weibo, the total number of views for topics related to Japan’s announcement of of nuclear contaminated water discharge exceeded 10 billion, and the incident’s influence index reached 93.5%. These statistics indicate that the incident has triggered intense public discussion and ensure that the study has access to a wealth of empirical data. Additionally, to ensure the comprehensiveness of the data, the sampling window was set from August 22, 2023 to September 5, 2023. This period captures the critical phase of public opinion formation. August 22 is the date of the official announcement, which served as the trigger for information dissemination. According to statistics from the Zhiwei Shijian platform (https://ef.zhiweidata.com), the following week marked the peak of the discussion, with the majority of original posts, comments, and shares appearing during this period. By September 5, the number of new posts per day had stabilized at a low level, indicating that the core discourse had largely settled. Therefore, this window covers the entire public opinion cycle of the event and provides comprehensive data for research.

Data collection was performed on Weibo (www.weibo.com) using a Python based crawler with the requests library. The crawler used a standard browser User Agent and a valid login Cookie to maintain session stability. A random delay of 3 seconds was introduced between page requests to avoid sending excessive traffic. Using Python language, with “nuclear contaminated water” as the key word and the time range set from August 22, 2023 to September 5, 2023, data collection was carried out on Weibo (www.weibo.com), resulting in 61,027 original Weibo posts. The collected data include user name, user authentication type, content of the post, number of likes, number of comments, and number of forwards. Additionally, this study strictly adhered to ethical principles, including authenticity, impartiality, openness and transparency, as well as data anonymization. All data used in this study are publicly available on Weibo and were processed solely for academic research. To protect user privacy, personally identifiable information was anonymized, and only the verification type of users was retained. A detailed description of the data collection and processing procedures was also provided to ensure transparency and reproducibility. The authors confirm that the data were collected from publicly accessible Weibo pages and processed in accordance with ethical principles of academic research. The collection method collection method did not circumvent any access restrictions or authentication mechanisms.

Subsequently, data cleaning was performed, including: ①deleting error, non-Chinese content data; ②removing data that are duplicated or irrelevant to the topic; ③eliminating URLs and emojis from the content. After cleaning, the final sample consisted of 56,663 posts, representing over 92% of all original posts that matched the keyword within the time window. To verify the validity of the cleaning process, three independent research assistants conducted a random sampling check. Each assistant manually reviewed a random sample of 400 posts and agreed that the cleaned data accurately preserved the original content. Therefore, the sample is considered highly representative of the public discourse on this event.

### 3.2. Operationalization of variables

#### 3.2.1. Dependent variables: likes, comments and forwards.

This study examines information behavior from three perspectives: liking, commenting, and forwarding. Taking the forwarding as an example, when people are interested in the information content and have the willingness to share it, they tend to participate in the dissemination of public opinion directly through forwarding. Based on this, forwards were measured by using the number of forwards of each post. However, forwarding is the main means of public opinion dissemination, but it cannot be used as the only criterion for information behavior [47].

Likes, comments and forwards are often viewed as indicators of the dissemination effect of public opinion on social media [48–50]. Likes indicate the degree of fulfillment of people’s information needs, and comments indicate people’s opinions and attitudes towards posts. Information and other exogenous factors not only promote people’s forwarding of information, but also have a significant impact on people’s liking and commenting behavior [51]. Consequently, a quantitative approach was adopted to examine multiple information behaviors on social media. Specifically, popularity was operationalized as the number of likes per post, while engagement was measured through comment counts per post.

#### 3.2.2. Information topic.

In the process of public opinion dissemination, information topics may differentiate and evolve due to cognitive differences among people. An interesting or discussion-worthy topic can stimulate users’ desire to participate and motivate them to generate a variety of information behaviors. On the contrary, when the topic of information is vague or unattractive, people tend to maintain a questioning and wait-and-see attitude, making it difficult to generate interactive behavior. Therefore, delineating information topic can help to study the role of information attributes in influencing information behavior.

Latent Dirichlet Allocation (LDA) model represents a three-tiered Bayesian topical paradigm. It unearths the concealed topic-related details in text via an unsupervised learning approach. Previous studies have proved that the LDA model can effectively identify the topic of the document [52–54]. This study employed LDA topic identification model to identify latent topics in the process of public opinion dissemination. The implementation process rigorously follows these stages: Firstly, data processing was performed using the jieba tokenization package to segment the original text data. By combining Harbin Institute of Technology’s stopword list, frequently occurring meaningless words identified through statistical analysis, and domain-specific terms such as URLs, emojis, and punctuation marks, a custom stopword list suitable for this study was created to remove meaningless segments. Next, an LDA topic identification model was trained and constructed. Specifically, this study used the Gensim development package to train LDA models and calculate the log-perplexity for a range of 2–10 topics. It is known that when the perplexity is low and the number of latent topics is minimal, the model exhibits strong sample prediction capabilities, and the clustering results are locally optimal [54]. The results of the LDA model training show that the model’s perplexity generally increases as the number of topic increases, but a local minimum is observed when the number of topics is 4. Therefore, this paper determines that the optimal number of topics is 4. Finally, information topics were uncovered by identifying high-probability words as their representations, while determining topic affiliation for each post.

#### 3.2.3. Emotional response.

Scholars have found that information containing emotional words are more likely to promote the dissemination of public opinion, and that containing different emotions has different roles in information dissemination [3,36]. Therefore, by calculating the scores of various types of emotions in the text, the influence of emotions on public opinion dissemination can be further investigated [28,55].

In recent years, scholars have explored the opinions and attitudes of the public through emotional dictionaries or machine learning methods [56]. It is noteworthy that emotion analysis differs from sentiment analysis. Rather than simply categorizing emotions as positive or negative, it subdivides them into several different types of emotions. For example, the existing mature Affective lexicon ontology of Dalian University of Technology classifies emotions into 7 major categories and 21 subcategories, and sets initial intensity levels of 1, 3, 5, 7, and 9, providing a rich framework for emotion analysis. In this study, emotion scores computation were computationally derived using the jieba tokenization package and Affective Lexicon Ontology. When posting, users often use different types of emotional words simultaneously, and all the emotional words in the posts may all contribute to the dissemination of public opinion. Therefore, instead of using emotion as a categorical variable, this study calculated the scores of all emotions in the posts.

#### 3.2.4. User credibility.

On Weibo, user credibility is operationalized through the platform’s official verification system. This system classifies accounts into four hierarchical types based on their identity authenticity and social influence: Blue V certified users representing official organizations, Gold V certified users representing particularly active users, Orange V certified users representing active users, and un-certified ordinary users. Prior studies have confirmed that verified users are perceived as more credible than unverified ones because identity validation reduces anonymity [46,57]. The verification type directly shapes the perceived credibility of the information source, which in turn influences the reach and dissemination effect of their posts [29,38]. Thus, user credibility was quantified through a point-based system: Blue V certified users receiving 5 points, Gold V certified users receiving 4 points, Orange V certified users receiving 3 points, and un-certified users receiving 0 points.

#### 3.2.5. Control variables.

Apart from the variables mentioned above, information richness, whether the post mentions someone else, the number of hashtags, the presence of URLs, and the length of text have the potential to influence the public’s information behavior and the dissemination of opinion. Therefore, in order to weaken the interference pattern formed by the confounding factors and to make the final results more credible and stable, the above variables are used as control variables to be integrated into the regression equation to carry out fine analysis.

The final variable definitions are shown in Table 1.

**Table 1:** Measurement of variables.

Variable name	Measure item	Description
*Dependent variables*		
Likes	Likes	Number of likes of each post.
Comments	Comments	Number of comments on each post.
Forwards	Forwards	Number of forwards of each post.
*Independent variables*		
Information topic	Topics	Posts are divided into 4 topics.
Anger	Anger	Anger score of each post.
Disgust	Disgust	Disgust score of each post.
Fear	Fear	Fear score of each post.
Good	Good	Good score of each post.
Sadness	Sadness	Sadness score of each post.
Surprise	Surprise	Surprise score of each post.
Happy	Happy	Happy score of each post.
*Moderate variable*		
User credibility	User credibility	Users are authenticated to 4 types
*Control variables*		
Richness	Richness	The richness level of each post.
Mention	Mention	Whether the post mentions someone else
Hashtag	Hashtag	Number of hashtags of each post
URL	URL	Whether the post has a URLs
Length	Length	Content length of each post

### 3.3. Descriptive analysis

After completing the data screening, this study performed a descriptive analysis of the variables (as shown in Table 2). It was found that people actively participated in information dissemination and emotional interactions during the event of Japan’s announcement of nuclear contaminated water discharge, generating a large amount of information behaviors. In terms of the types of information behavior, people were more inclined to use simple likes to express their personal opinions than comments and forwards.

**Table 2:** Descriptive analysis.

Variable	Max	Min	Percentage (%)/Mean (S.D.)
Likes	460703	0	81.181 (2603.458)
Comments	13110	0	5.397 (94.530)
Forwards	15237	0	4.807 (119.629)
*Topics*			
Topic 1			1.850
Topic 2			8.822
Topic 3			13.286
Topic 4			76.043
Anger	14	0	6.127/0.080 (0.368)
Disgust	130	0	50.229/1.440 (3.055)
Fear	38	0	14.032/0.189 (0.610)
Good	75	0	45.081/1.327 (2.917)
Sadness	26	0	17.308/0.290 (0.858)
Surprise	6	0	2.370/0.028 (0.195)
Happy	18	0	18.755/0.319 (0.912)
User credibility	5	0	1.656 (2.019)
Richness	3	1	1.531 (0.734)
Mention	1	0	0.060 (0.238)
Hashtag	22	0	1.107 (1.247)
URL	1	0	0.049 (0.216)
Length	1596	1	38.535 (62.351)

In terms of content, people mainly focused on 4 topics in their discussions. Among them, topic 1 focuses on the import and export trade of Japanese aquatic products; topic 2 reveals the phenomenon of seawater variability caused by the nuclear contaminated water discharge. topic 3 is the exceeding of radioactivity of nuclear contaminated water. topic 4 discusses the impact of nuclear contaminated water discharge on countries around the world. Obviously, topics 2, 3 and 4 constitute a complete framework for the discussion of the “phenomenon - risk - impact” of the nuclear contamination incident, while topic 1 is an economic issue that is less related to the core event. There is a tendency to discuss the impact of nuclear contaminated water on the world (76.043%), which is likely due to its potential risks to the ecological environment and human health.

In terms of emotional response, people express more disgust towards this policy (50.229%). From the perspective of user credibility, ordinary users are the main participants in information dissemination. In terms of post content, posts with videos or images or posts with multiple hashtags are more likely to spread on social media. In addition, it was found that people were very willing to express their opinions and attitudes in this incident, so the posts were very long.

## 4. Results

### 4.1. Baseline regression

Due to the highly skewed distribution of the dependent variables and certain control variables, logarithmic conversion was applied to normalize the data and reduce heteroscedasticity [20]. In addition, Considering the possible problem of multicollinearity between the variables, the study uses likes, comments, and forwards as dependent variables to conduct collinearity tests. The test result that the variance inflation factor (VIF) values are all less than 3.5 indicates that there is no obvious multicollinearity problem among the variables, which meets the prerequisite for conducting regression analysis.

The study used information topic and emotional response as independent variables, and likes, comments and forwards as dependent variables to establish a benchmark regression model (the results are shown in Table 3). Models 1, 2 and 3 respectively test the influence of information topic on likes, comments and forwards. Since information topic is a categorical variable, topic 1 was used as a reference group to analyze the effect of different topics on information behavior. The results of Model 1 show that Topic 2 (β = 0.164, p < 0.001), Topic 3 (β = 0.271, p < 0.001), and Topic 4 (β = 0.333, p < 0.001) all had a significant positive effect on likes. H1a was supported. The results of model 2 and model 3 were similar to model 1. Topic 2, 3 and 4 all had a significant positive effect on comments and forwards. H1b and H1c were supported. The results demonstrate that information topics have the capacity to drive all types of informational behavior, and that topics directly related to the public opinion event (topics 2/3/4) have stronger driving force than fringe topics (topic 1).

**Table 3:** Main effect regression.

	(1)			(2)			(3)				(4)				(5)				(6)		
	Likes			Comments			Forwards				Likes				Comments				Forwards		
Topic 2	0.164***			0.063***			0.083***														
Topic 3	0.271***			0.109***			0.131***														
Topic 4	0.333***			0.138***			0.156***														
Anger											0.052***				0.017**				0.014**		
Disgust											0.001				0.004***				0.009***		
Fear											0.008				−0.003				−0.003		
Good											0.004**				0.003**				0.004***		
Sadness											−0.006				−0.006*				−0.010***		
Surprise											0.060***				0.047***				0.038***		
Happy											−0.002				−0.006*				−0.010***		
Richness	0.201***			0.148***			0.146***				0.194***				0.146***				0.146***		
Mention	−0.200***			−0.084***			−0.077***				−0.203***				−0.084***				−0.075***		
Hashtag	−0.009***			−0.005**			−0.004**				−0.009**				−0.005**				−0.003*		
URL	−0.282***			−0.125***			−0.072***				−0.267***				−0.117***				−0.062***		
Length	0.321***			0.150***			0.147***				0.216***				0.093***				0.074***		
Constant	−0.854***			−0.646***			−0.724***				−0.411***				−0.452***				−0.499***		
Observations	56663			56663			56663				56663				56663				56663		
Adjusted R^2^	0.085			0.080			0.098				0.080				0.079				0.099		

*Note.* Standard errors in parentheses; *p < 0.05, **p < 0.01, ***p < 0.001.

Meanwhile, risk communication theory suggests that hazards perceived as affecting large populations or having delayed, uncertain, or catastrophic consequences tend to attract greater public attention [58,59]. In the event of Japan’s announcement of nuclear contaminated water, the risk involves not only immediate ecological damage but also long term, transboundary health effects. The discharge of nuclear contaminated water from Japan implies that a variety of radioactive substances will spread through the oceans and into global waters, potentially causing unpredictable effects on the global marine environment and human health. Within the ELM framework, such high personal relevance and perceived threat increase users’ motivation to process information systematically, thereby amplifying the effect of central [11]. Consistent with these theoretical expectations, results show that the impacts of nuclear contaminated water discharge (Topic 4) attracted the highest volume of discussion and elicited the strongest behavioral responses across likes, comments and forwards.

Models 4, 5 and 6 respectively test the influence of emotional response on likes, comments and forwards. According to the ELM, people use their immediate emotional feelings as a shortcut for decision-making, with stronger emotions leading to faster response [13]. Anger had a significant positive effect on likes (β = 0.052, p < 0.001), comments (β = 0.017, p < 0.001) and forwards (β = 0.014, p < 0.001). Disgust had a significant positive effect on comments (β = 0.004, p < 0.001) and forwards (β = 0.009, p < 0.001). H2a, H3a, H4a, H3b and H4b were supported. Consistent with research on the spread of emotions, anger and disgust are the most immediate reactions people have to negative events, and they can trigger a strong desire to express oneself [60]. In contrast, the null effect of fear aligns with the defense cascade model, which posits that fear triggers freezing or passive avoidance rather than active behavioral engagement [61].

Good had a significant positive effect on likes (β = 0.004, p < 0.01), comments (β = 0.003, p < 0.01) and forwards (β = 0.004, p < 0.001). Surprise had a significant positive effect on likes (β = 0.060, p < 0.001), comments (β = 0.047, p < 0.001) and forwards (β = 0.038, p < 0.001). H2d, H3d, H4d, H2f, H3f, H4f were supported. On Chinese social media platforms, good and surprise are often strategically used to satirize negative events while evading the government’s public opinion control [20]. In the event of Japan’s announcement of nuclear contaminated water, good and surprise are associated with expectation violation, which attracts attention and motivates sharing. Sadness had a significant negative effect on comments (β = −0.006, p < 0.05) and forwards (β = −0.010, p < 0.001). Happy also had a significant negative effect on comments (β = −0.006, p < 0.05) and forwards (β = −0.010, p < 0.001). H3e, H4e, H3g, H4g were supported. The findings show that not all negative or positive emotions motivate information behavior equally. Emotional effects depend on specific emotion type, context congruence, and motivational tendency.

After comparing the results of all models, the study found that liking was most significantly influenced by the promotion of information topic and the promotion of emotional response, followed by forwarding, then commenting. From the ELM perspective, the tiered influence pattern reflects distinct information processing pathways. Liking, as a low-cognitive-effort behavior, aligns with peripheral route processing. When people feel empathy for a topic or emotion, the simple and quick act of liking is the preferred way to express it. Commenting demands central route processing, requiring systematic content analysis and argument construction. The high cognitive threshold reduces the impact of peripheral emotional cues, making it less responsive to emotional appeals. Forwarding represents a hybrid of both pathways: while emotional cues trigger initial sharing motivation, users also evaluate content relevance before forwarding.

### 4.2. Moderating effects of user credibility

To further investigate whether disparities exist in the associations among information, emotion and information behavior, this study centered around user credibility as a pivotal component in the analytical framework. It aimed to further scrutinize how user credibility

Table 4 shows the manner in which user credibility moderates the influence of information topic upon information behavior. Models 1, 2, and 3 respectively represent the effects of user credibility moderating information topic on likes, comments, and forwards. In Model 1, the interaction term between user credibility and topic 2 (β = 0.029, p < 0.01), that between user credibility and topic 3 (β = 0.066, p < 0.001), as well as that between user credibility and topic 4 (β = 0.060, p < 0.001) exerted a notably positive influence on likes. Thus, user credibility served to positively modulate the correlation between information topics and the act of liking. H5a was supported. The results of model 2 and model 3 were similar to model 1. The interaction terms between user credibility and topic 2, topic 3 and topic 4 exerted a notably positive influence. Thus, user credibility had a different effect on the relationship between information topics and comments or forwards. H5b and H5c were supported.

**Table 4:** Moderating effect of user credibility on the information topics.

	(1)			(2)			(3)		
	Likes			Comments			Forwards		
Topic 2	0.124***			0.047**			0.069***		
Topic 3	0.197***			0.077***			0.101***		
Topic 4	0.299***			0.129***			0.149***		
User credibility	0.060***			0.035***			0.037***		
Topic 2 * User credibility	0.029**			0.012^+^			0.010^+^		
Topic 3 * User credibility	0.066***			0.029***			0.029***		
Topic 4 * User credibility	0.060***			0.027***			0.026***		
Richness	0.135***			0.108***			0.106***		
Mention	−0.207***			−0.088***			−0.081***		
Hashtag	−0.010***			−0.006***			−0.005***		
URL	−0.234***			−0.096***			−0.042***		
Length	0.313***			0.145***			0.142***		
Constant	−0.799***			−0.625***			−0.704***		
Observations	56663			56663			56663		
Adjusted R^2^	0.107			0.098			0.123		

*Note.* Standard errors in parentheses; + p < 0.1, *p < 0.05, **p < 0.01, ***p < 0.001.

moderates the effects of information topic and emotional response on information behavior.

**Fig 2:**
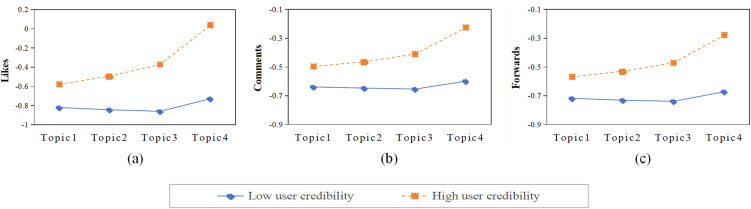
Moderating effect of user credibility on the information topics.

Graphical methods were used to illustrate the moderating effect of user credibility in the relationship between information topics and information behaviors. The results are shown in Fig 2. Compared to Topic 1, the remaining three topics all show an enhanced promotion effect on likes, comments, and forwards as user credibility increases. It becomes evident that the moderating function of user credibility within the association between information topics and comments exhibits a striking similarity to that in the relationship between information topics and forwards. However, when it comes to the relationship between information topics and likes, the moderating force of user credibility proves to be considerably stronger. In addition, substantial disparities exist in the spreading patterns of posts among users possessing varying degrees of credibility across the four topics. In particular, when discussing the impact of nuclear contaminated water on the world, the distinctions in the dissemination of information by users with diverse credibility levels become strikingly apparent.

Table 5 demonstrates how user credibility moderates the effect of emotional response on information behavior. Models 1, 2, and 3 respectively represent the effects of user credibility moderating emotional response on likes, comments, and forwards. Where, the interaction terms between user credibility and anger (β = 0.037, p < 0.001), disgust (β = 0.006, p < 0.001), fear (β = 0.018, p < 0.001), good (β = 0.006, p < 0.001), sadness (β = 0.013, p < 0.001), surprise (β = 0.040, p < 0.001), and happy (β = 0.017, p < 0.001) in model 1 all exerted a notably positive influence on likes. H5d was supported. The results of model 2 and model 3 were similar to the results of model 1, with significant positive effects of user credibility and the interaction terms of the seven different emotions on both comments and forwards. Thus, user credibility served to positively modulate the correlation between emotional response and comments and forwards. H5e, H5f were supported.

**Table 5:** Moderating effect of user credibility on the 7 emotional responses.

	(1)			(2)			(3)		
	Likes			Comments			Forwards		
Anger	0.047***			0.020***			0.021***		
Disgust	0.003*			0.004***			0.008***		
Fear	0.014**			0.003			0.007*		
Good	0.004**			0.004***			0.006***		
Sadness	0.005			0.003			0.003		
Surprise	0.065***			0.050***			0.045***		
Happy	0.000			0.001			0.002		
User credibility	0.056***			0.034***			0.035***		
Anger * User credibility	0.037***			0.015***			0.018***		
Disgust * User credibility	0.006***			0.003***			0.004***		
Fear * User credibility	0.018***			0.006***			0.010***		
Good * User credibility	0.006***			0.002***			0.003***		
Sadness * User credibility	0.013***			0.007***			0.008***		
Surprise * User credibility	0.040***			0.022***			0.025***		
Happy * User credibility	0.017***			0.006***			0.007***		

*Note.* Standard errors in parentheses; *p < 0.05, **p < 0.01, ***p < 0.001.

**Fig 3:**
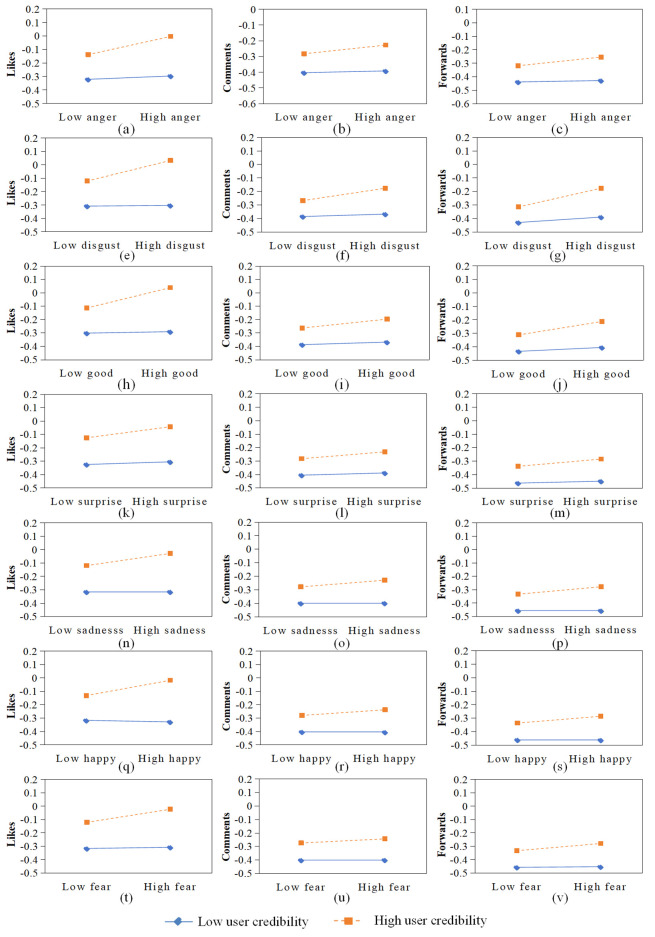
Moderating effect of user credibility on the emotional responses.

To further clarify the moderating effect of users with different credibility in the relationship between emotion and information behavior, the study further plotted the moderating effect (Fig 3). Among the seven emotions, the posts published by users with high credibility will receive more likes, comments and forwards than those published by users with low credibility. It can be found that in the scenarios involving the interplay between anger and comments, and also between anger and forwards, user credibility’s moderating capacity appears to be rather limited, while the moderating efficacy of user credibility in anger and likes is notably more prominent. The situation is the same in the other six emotions.

Among the 4 topics or the 7 emotions, the posts published by users with high credibility will receive more likes, comments and forwards. This indicates that the moderating function played by user credibility is a common phenomenon. From an ELM perspective, user credibility acts as a powerful peripheral cue that amplifies the effects of both central information cuesand peripheral emotional cues. When users are considered highly trustworthy, people may place unwarranted trust in their posts. Specifically, when users perceive that the information publisher has high credibility, they are more inclined to believe the information content and are more willing to participate in the dissemination of the information.

## 5. Discussion

Drawing on social media data, this study integrates ELM to examine how information topic and emotional response shape liking, commenting and forwarding, with user credibility as a moderator. Unlike prior unidimensional or binary-sentiment research, we offer three advances. First, engagement follows a cognitive effort gradient: liking is most sensitive to both cues, commenting least. Second, emotions have heterogeneous effects: anger and disgust promote information behavior, while sadness and happy suppress it and fear shows no effect. Third, user credibility moderates amplifying both central and peripheral effects. These findings extend ELM and source credibility theory.

### 5.1. Theoretical implications

Firstly, while previous studies have applied ELM to understand information dissemination, most have treated user engagement as a monolithic conceptor have aggregated likes, comments, and forwards into a single information behavior. In contrast, this study explicitly distinguishes these three behaviors based on their cognitive effort demands, showing that likesare most sensitive to both information topics and emotional responses, while commentsare least sensitive. This study built on previous research by incorporating likes and comments into the analysis model in order to more fully understand users’ attitudes and behavioral patterns in the process of public opinion dissemination.

Secondly, the study considered more emotion dimensions and focused on all emotion scores in the post, providing a more comprehensive and objective perspective on emotion analysis. Previous studies have been devoted to teasing out the differential impacts of positive and negative emotions during the course of information dissemination [20,35,62]. However, all emotional words may promote information behavior [3]. By distinguishing specific emotional dimensions, we can identify the varying effects of different emotions on informational behavior [63]. Based on this, the study focused on the different emotion scores in each post, which further validates and complements the effect of emotions on information behavior.

Finally, the study took user credibility as a moderating variable to test the differences in the influence of information attributes on information behavior. Previous studies have classified users into different types such as government user, media user, and ordinary user according to their nature, and observed the differences in the influence of user type on the relationship between information attributes and dissemination effects [46]. However, the development of new media has broken the information monopoly of traditional media, and people have elected their own leaders outside the mainstream media. Many certified active users on the Weibo gain public trust through interaction [57]. In this investigation, identity certification on social media was used as a standard for classifying user credibility. The study analyzed how people make behavioral choices based on user identity, and the results contribute to a more profound understanding of the moderating effects of user credibility, significantly enriching the theory of user credibility.

### 5.2. Practical implications

The results have practical guiding significance in enhancing the public opinion guidance ability and risk response ability of government organizations. Firstly, it was found that user engagement can be increased by setting valuable and engaging topics. To promote the effect of information dissemination, it is necessary to purposefully enhance the value content of the topic to stimulate the user’s desire to share respective information. Secondly, the study has confirmed that in crisis situations, negative emotions have an inherent advantage of spreading and are more likely to stimulate people’s desire to share. Authorities can use the existing negative emotion information to increase people’s crisis awareness and risk perception sensitivity. Focusing on the scores of various emotions in the posts, they can also predict people’s behavioral responses in public opinion events. However, placing too much emphasis on highly relevant topics or relying too heavily on negative emotional appeals may inadvertently heighten public anxiety or lead to information fatigue. Therefore, it is recommended to adopt a balanced approach that combines risk communication with actionable guidance. In addition, the study found that people are more willing to pay attention to and share information posted by highly credible users. By encouraging head users in social media to post positive and interesting information, it can attract users’ attention and participation, thus effectively monitoring the direction of public opinion.

### 5.3. Limitations and directions for future research

The study still has some limitations. Firstly, the data acquisition platform and the type of case events are relatively limited. The data were collected from a single event on Weibo, which has strong geopolitical salience in China. Cultural context limits generalizability to other countries or platforms with different systems. Future studies will consider data collection for different media platforms and multiple public opinion events to augment data resources and extend the general applicability of the study’s outcomes. Secondly, the study achieved emotion analysis with the help of the affective lexicon ontology of Dalian University of Technology, which may not be precise enough due to the lack of pertinence in the field of events. However, the ambiguity and ironic expression of netizens may lead to errors in the judgment of emotional tendency. The language processing methods will be gradually optimized in the future to provide more accurate and reliable emotion analysis results for practical application scenarios.

## 6. Conclusion

This study integrates the elaboration likelihood model with behavioral stratification (likes/comments/forwards) to decode how information topic and emotional response guide public opinion under the moderation of user credibility. 56,663 Weibo posts were used for empirical analysis, and it found that information topic and specific emotions (anger and disgust) significantly increase information behavior. User credibility consistently moderates these relationships, and liking is the most sensitive behavior. These findings provide a basis for authorities to predict trends in the spread of public opinion. Authorities may prioritize high credibility users, highlight impact relevant topics, and monitor specific negative emotionsto anticipate public engagement. However, these practical recommendations must be applied cautiously, considering cultural specificity, platform differences and potential side effects such as panic or distrust.
